# Substantial Extracellular Metabolic Differences Found Between Phylogenetically Closely Related Probiotic and Pathogenic Strains of *Escherichia coli*

**DOI:** 10.3389/fmicb.2019.00252

**Published:** 2019-02-19

**Authors:** Justin J. J. van der Hooft, Robert J. Goldstone, Susan Harris, Karl E. V. Burgess, David G. E. Smith

**Affiliations:** ^1^Bioinformatics Group, Wageningen University, Wageningen, Netherlands; ^2^The Francis Crick Institute, London, United Kingdom; ^3^Institute of Biological Chemistry, Biophysics and Bioengineering, Heriot-Watt University, Edinburgh, United Kingdom; ^4^Glasgow Polyomics, College of Medical, Veterinary and Life Sciences, University of Glasgow, Glasgow, United Kingdom

**Keywords:** *Escherichia coli*, Nissle 1917, probiotic, pathogenic, extracellular metabolome, mass spectrometry, metabolomics

## Abstract

Since its first isolation a century ago, the gut inhabitant *Escherichia coli* strain Nissle 1917 has been shown to have probiotic activities; however, it is yet not fully elucidated which differential factors play key roles in its beneficial interactions with the host. To date, no metabolomics studies have been reported investigating the potential role of small molecules in functional strain differentiation of Nissle from its genetically close neighbors. Here, we present results of liquid chromatography coupled to high-resolution mass spectrometry characterization of extracellular metabolomes of *E. coli* strains as a proxy of their bioactivity potential. We found that phylogroup B2 strains exported a more diverse arsenal of metabolites than strains of other phylogroups. Zooming into the phylogroup B2 metabolome identified consistent substantial differences between metabolic output of *E. coli* Nissle and other strains, particularly in metabolites associated to the Argimine biosynthesis pathway. Nissle was found to release higher levels of Ornithine and Citrulline whilst depleting greater amounts of Arginine from the medium. Moreover, a novel Nissle-specific metabolite not reported before in bacteria, 5-(Carbamoylamino)-2-hydroxypentanoic acid (Citrulline/Arginic Acid related) was observed. Finally, Nissle, CFT073 and NCTC12241/ATCC25922 shared the excretion of N5-Acetylornithine, whereas other strains released N2-Acetylornithine or no N-Acetylornithine at all. Thus, we found substantial metabolic differences in phylogenetically very similar *E. coli* strains, an observation which suggests that it is justified to further investigate roles of small molecules as potential modulators of the gut environment by probiotic, commensal, and pathogenic strains, including *E. coli* Nissle 1917.

## Introduction

*Escherichia coli* is a highly diverse and versatile bacterial species many representatives of which are common resident organisms of the intestinal tract in homeothermic animals. *E. coli* can be classified on the basis of many characteristics including their ability to cause disease, frequently referred to as “pathotype” (Robins-Browne et al., 2016). These include intra-intestinal pathogenic (InPEC) and extra-intestinal pathogenic (ExPEC) strains, exemplified respectively, by enterohaemorrhagic *E. coli* (EHEC such as O157:H7) and uropathogenic *E. coli* (UPEC). Although *E. coli* strains include significant pathogenic strains, the majority of *E. coli* co-exist with their warm-blooded hosts in commensal or mutualistic relationships. In addition, some *E. coli* strains have been shown to possess overtly beneficial activities and have been exploited as probiotic organisms (Wassenaar, 2016). Among these are *E. coli* strain Nissle 1917 (henceforth referred to as Nissle) which has received much attention to characterize its probiotic activities (Trebichavsky et al., 2010; Alvarez et al., 2016; Sonnenborn, 2016; Wassenaar, 2016).

From applied and fundamental studies of Nissle it is evident that its probiotic capacity is multi-faceted and involves competition with pathogens (Huebner et al., 2011; Deriu et al., 2013; Sassone-Corsi et al., 2016), promotion of mucosal integrity (Hering et al., 2014; Wang et al., 2014), modulation of intestinal innate defenses (Zyrek et al., 2007; Guzy et al., 2008; Mondel et al., 2008), bioavailability of enteroendocrine effectors (Nzakizwanayo et al., 2015) and modulation of peristaltic contractions (Bär et al., 2009; Dalziel et al., 2015). These activities correlate with the usage of this strain in amelioration of acute and chronic enteric diseases (Henker et al., 2008; Kruis et al., 2012; Losurdo et al., 2015; Scaldaferri et al., 2016) and prevention and treatment of infectious diseases (Kandasamy et al., 2017). In addition, Nissle and other probiotic bacteria may exert benefits beyond the intestinal tract (Mazzoli and Pessione, 2016).

Intriguingly, Nissle is phylogenetically closely-related to clinically significant pathogenic strains, specifically uropathogenic (UPEC) and other invasive (ExPEC) strains (Dobrindt et al., 2010; Hancock et al., 2010; Vejborg et al., 2010). Despite sharing many virulence factors (including adhesins, toxins and effectors) with these related strains, Nissle is clearly distinct in its interactions with host tissues although the factors responsible remain incompletely characterized (Becker et al., 2014). Much attention has focussed on macromolecular structures [e.g., surface polysaccharides (Hafez et al., 2010) and protein components such as adhesins and flagella (Schlee et al., 2007)], and toxins. Besides siderophores involved in iron acquisition, there has been less emphasis towards small extracellular molecule components and their potential role in bacterium-host interaction for Nissle.

The role of small molecules in bacterium-host interactions has been an area of increasing research interest over recent years (Donia and Fischbach, 2015). Whilst Nissle is the most-studied probiotic since its discovery 100 years ago (Sonnenborn, 2016; Wassenaar, 2016), its ‘core metabolism’ and the metabolites (other than siderophores) Nissle releases into its environment have been largely overlooked so far. In this study, a metabolomic survey of Nissle alongside other *E. coli* strains from B2 and other phylogroups was performed. Since the B2 phylogroup strains contained a high and unique metabolite diversity, we postulated that *E. coli* Nissle 1917 (i) could possess metabolic characteristics that distinguish it from other *E. coli* including phylogenetically closely-related pathogenic strains and that (ii) these components could contribute towards probiotic activities of Nissle. Towards this goal, we used high-resolution LC-MS and LC-MS/MS approaches to characterize extracellular metabolomes in a subset of *E coli* B2 phylogroup strains phylogenetically very close to Nissle, followed by identifications and annotations of metabolites distinct for Nissle. Our observations showed that Nissle exports distinct Arginine biosynthesis-related metabolites, namely Citrulline, a Citrulline analog, and Ornithine, whilst Arginic (Argininic) acid was released by its close relatives. These results were validated through an independent experimental study with extracellular samples from these *E. coli* strains. This work underlines that high-resolution mass spectrometry analyses combined with fragmentation approaches are capable of accurately characterizing bacterial metabolites and thus molecular bacterial phenotypes. We conclude that small molecule extracellular profiles show substantial differences between the probiotic and pathogenic strains and that metabolites and yet undescribed metabolic pathways may contribute to as yet undiscovered unforeseen activities in bacterium-host interactions.

## Materials and Methods

### Bacterial Isolates, Culture and Sample Preparation

*Escherichia coli* strains MG1655, HS, TUV93-0, 042, O103:H2, APEC O1, ABU83972, J96, 536, NCTC12241/ATCC25922 (this strain is known under these two identifiers and will be denoted as such throughout this study), CFT073 and Nissle 1917 were all stored as glycerol stocks at -80°C (see Supplementary Table S1 for further details of strains). Stocks were revived by culture on LB agar plates. Single colonies (in separate replicate culture vessels) were cultured in DMEM (Low glucose [D6046], Sigma-Aldrich) overnight at 37°C without shaking. Subsequently, each culture was diluted in DMEM to OD_600_ 0.01 and grown to an OD_600_ of 0.8 (approximately 7 h). Cultures were quenched in a dry ice/ethanol bath then centrifuged at 4000 ×*g* at 4°C for 10 min. Supernatant from each culture was recovered after centrifugation, passed through a sterilization filter (0.2 μm), and mixed 1:4 with chloroform/methanol (1:3). Triplicate samples of fresh media were mixed 1:4 with chloroform/methanol (1:3). All samples were incubated for 1 h at 4°C with agitation then centrifuged at 13,000 g for 3 min at 4°C and solvent extracts recovered and stored at -80°C until mass spectrometry. Solvent extracts (1:3:1 chloroform:methanol:water) of cells from selected strains were as reference material for LC-MS/MS-based metabolite annotations. Two fresh solvent samples were also collected as solvent controls.

### Genomic Analyses

Chromosome sequences for the *E. coli* B2 strains were sourced directly from NCBI and have the following assembly designations: CFT073 (GCA_000007445.1), ABU83972 (GCA_000148365.1), 536 (GCA_000013305.1), NCTC12241/ATCC25922 (GCA_000743255.1), Nissle 1917 (GCA_003546975.1), and J96 (GCA_000295775.2). The genome sequence for strain MG1655 (GCA_000005845.2) was also sourced for reference purposes. A strain-versus-strain comparison of complete genome sequences was carried out using the blastn tool with default settings. Also, all annotated protein sequences for *E. coli* CFT073 were used to search for matches in ABU83972, 536, NCTC12241/ATCC25922, Nissle 1917, J96, and MG1655 using blastp to return “% sequence cover” and “% sequence identity” results for every gene. Equivalence cut-off was set as ≥ 90% sequence cover and identity.

### Chemicals

HPLC-grade methanol, acetonitrile, and analytical reagent grade chloroform were acquired from Fisher Scientific, Loughborough, United Kingdom. HPLC grade H_2_O was purchased from VWR Chemicals, Fountenay-sous-Bois, France. Ammonium carbonate was acquired from Fluka Analytical (Sigma Aldrich), Steinheim, Germany. Arginic acid was obtained from Sigma Aldrich.

### Study Setup

For the initial study (Study 1), triplicate cultures were grown and both whole cell extracts and supernatant were analyzed with LC-MS. Selected samples of Study 1 were fragmented separately. For the B2-focussed study (Study 2) and Validation Study (Study 3), five replicate cultures were grown and analyzed. In addition, the five replicates were pooled to generate LC-MS/MS fragmentation data for each strain at the same time as full scan MS data generation.

### Analytical Instrumentation and Settings

#### Chromatography

The samples were analyzed using a Thermo Scientific Ultimate 3000 RSLCnano system (Thermo Scientific, CA, United States). The pHILIC separation was performed with a SeQuant ZIC-pHILIC column (150 × 4.6 mm, 5 μm) equipped with the corresponding pre-column (Merck KGaA, Darmstadt, Germany) – the column temperature was maintained at 25°C. A linear biphasic LC gradient was conducted from 80% B to 20% B over 15 min, followed by a 2 min wash with 5% B, and 8 min re-equilibration with 80% B, where solvent B is acetonitrile and solvent A is 20 mM ammonium carbonate in water. The flow rate was 300 μL/min, column temperature was held at 25°C, injection volume was 10 μL, and samples were maintained at 4°C in the autosampler (Creek et al., 2011).

#### Mass Spectrometry

##### Study 1 – *E. coli* B2 and other phylotypes

The LC system was coupled to a Thermo Scientific Exactive Orbitrap mass spectrometer equipped with a HESI II interface (Thermo Scientific, Hemel Hempstead, United Kingdom). The set up was calibrated (Thermo calmix) in both ionization modes and tuned for the lower m/z range before analysis. Full scan (MS1) data was acquired in positive and negative switching mode in profile mode at 50,000 resolution (at m/z 200) using 1 microscan, an AGC target of 10^6^ cts, a maximum injection time of 250 ms, with spray voltages +4.5 and -3.5 kV, capillary temperature 275°C, heater temperature of 150°C, sheath gas flow rate 40 a.u., auxiliary gas flow rate 5 a.u., sweep gas flow rate 5 a.u, a full scan mass window of 70–1400 m/z, and using m/z 74.0964 (+) and m/z 112.98563 (-) as locking masses.

##### Studies 2 & 3 – B2-focussed and validation Study

The LC system was coupled to a Thermo Scientific Q-Exactive Orbitrap mass spectrometer equipped with a HESI II interface (Thermo Scientific, Hemel Hempstead, United Kingdom). The set up was calibrated (Thermo calmix) in both ionization modes and tuned for the lower m/z range before analysis. Full scan (MS1) data was acquired in positive and negative switching mode in profile mode at 35,000 resolution (at m/z 200) using 1 microscan, an AGC target of 10^6^ cts, a maximum injection time of 250 ms, with spray voltages +3.8 and -3.0 kV, capillary temperature 320°C, heater temperature of 150°C, sheath gas flow rate 40 a.u., auxiliary gas flow rate 5 a.u., sweep gas flow rate 5 a.u, a full scan mass window of 70–1050 m/z, and using m/z 74.0964 (+) and m/z 112.98563 (-) as locking masses. LC-MS/MS fragmentation data of selected samples from Study 1 were also obtained on this setup using similar settings.

### Mass Spectrometry Fragmentation

Fragmentation data (LC-MS/MS) was obtained in positive and negative ionization combined and separate fragmentation modes as described in van der Hooft et al. (2016). Briefly, for separate mode, a duty cycle consisted of one full scan (MS1) event and one Top5 (or Top10) MS/MS (MS2) fragmentation event, with full scan (MS1) resolution (at m/z 200) was set to 70,000, the AGC target set to 1 × 10^6^, and the maximum injection time set to 120 ms. MS/MS (MS2) resolution (at m/z 200) was set to 17,500, the AGC target set to 2 × 10^5^, MS/MS maximum injection time was set to 80 ms and the underfill ratio was set to 10%, with a resulting intensity threshold of 2.5 × 10^5^ cts. For combined mode, a duty cycle consisted of two of the above events in positive and negative ionization mode with the following modifications: full scan (MS1) resolution (at m/z 200) was set to 35,000, MS/MS resolution was set to 35,000, the AGC target was set to 1 × 10^5^, and the maximum MS/MS filling time was set to 120 ms with an underfill ratio of 20%, resulting in an intensity threshold of 1.7 × 10^5^. Further settings were as specified for full scan analysis above.

### Data Acquisition

Blank runs, quality control samples (beer and serum extracts in accordance with standard procedures at Glasgow Polyomics) to assess the performance of the mass spectrometer in terms of chromatography and mass intensities, and three standard mixes containing 150 reference compounds available from Glasgow Polyomics were run to assess the quality of the mass spectrometer and to aid in metabolite annotation and identification (Creek et al., 2011). Pooled sample containing all samples was run prior to and across the batch every 6th sample to monitor the stability and quality of the LC–MS run, whereas the samples were run in a randomized order. Thermo Xcalibur Tune software (version 2.5) was used for instrument control and data acquisition.

Immediately after acquisition, all raw files were converted into mzXML format, thereby centroiding the mass spectra and separating positive and negative ionization mode spectra into two different mzXML files using the command line version of MSconvert (ProteoWizard). Data quality assurance was performed and accurate masses of standards were obtained well within 3 ppm accuracy and intensities of the quality control samples (a beer extract and a serum extract) were within specifications.

### Data Processing and Analysis

MzXML files were uploaded into IDEOM (Creek et al., 2012) (Study 1) and PiMP^1^ (Studies 2 & 3). Both workflows rely on XCMS (Smith et al., 2006) and MzMatch (Scheltema et al., 2011) for LC-MS peak picking and alignment across samples. IDEOM results in an Excel file that contains the results with PiMP being a web-based tool.

### Data Availability

All MzXML files used for this study were uploaded into the GNPS repository (Wang et al., 2016) and can be accessed through here: ftp://massive.ucsd.edu/ completed with for Study 1: MSV000081287, Study 2 MSV000081288, and Study 3 MSV000081289. Each data set consists of both full scan and fragmentation data folders with each of them containing positive ionization mode and negative ionization mode data folders.

### Metabolite Annotation

Metabolites that matched to a standard and which fragmentation spectrum matched with that of reference MS/MS spectra were considered identified [Metabolite Identification Level 1 according to the Metabolomics Standards Initiative (MSI) (Sumner et al., 2007)]. If no such match was found, fragmentation spectra were analyzed and compared to reference spectral databases, i.e., using MzCloud ^2^ and Metlin^3^ (Tautenhahn et al., 2012), and analyzed using MaGMa ^4^ (Ridder et al., 2013). Annotations resulting from those analyses reported in this study are all of MSI level 2.

## Results

### Metabolic Capacities of B2 Strains versus Non-B2 Strains

**FIGURE 1:**
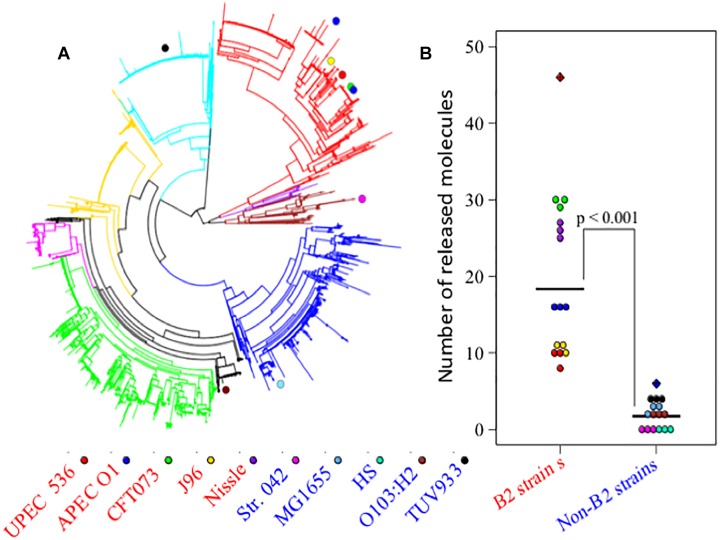
**(A)** *Escherichia* *coli* phylogenetic distance of *E. coli* strains used in Study 1 (*E. coli* B2 and other phylotypes study) with each strain represented by a colored circle – notice the tight grouping of the B2 strains in the upper right segment of the circle. **(B)** Numbers of released metabolites by phylogroup B2 strains that were not found in non-B2 strains and vice versa following the same strain labeling as for **(A)**. The results show that B2 strains possess a more diverse arsenal of small molecules than the non-B2 strains measured in this study. In total, phylogroup B2 strains biosynthesise 46 metabolites that are not produced by non-B2 strains (red hatched diamond), whereas non-B2 strains produce only 6 metabolites that could not be detected in the B2 group (blue hatched diamond).

The divergent gene content in the genomes of the B2 phylogroup is thought to be amongst the most varied of all the *E. coli* phylogroups (Touchon et al., 2009). To investigate if that could contribute to the production of a diverse set of small molecules, the metabolic capacities of 10 *E. coli* strains were initially assessed (Study 1). Five of those strains belong to phylogroup B2, whereas five other strains represented phylogroups A, B1, D, or E (see Figure 1A). Observation of the metabolite output in the extracellular metabolic profiles (Figure 1B) shows significant differences in the number of discriminative measured metabolites biosynthesised by the B2 compared with the non-B2 isolates (*p* value < 0.001). Some B2 strains, for example Nissle and CFT073, produced more than 20 metabolites which are not biosynthesised and released by any strain in the non-B2 group (Figure 1B) thus highlighting the diversity and uniqueness of B2 small molecule profiles. We focussed on metabolic profiles of B2 strains including probiotic and pathogenic strains to investigate whether differentiating metabolites might contribute to the differential phenotypes of those related strains.

### Profiling of Extracellular Metabolome of B2 Strains

**FIGURE 2:**
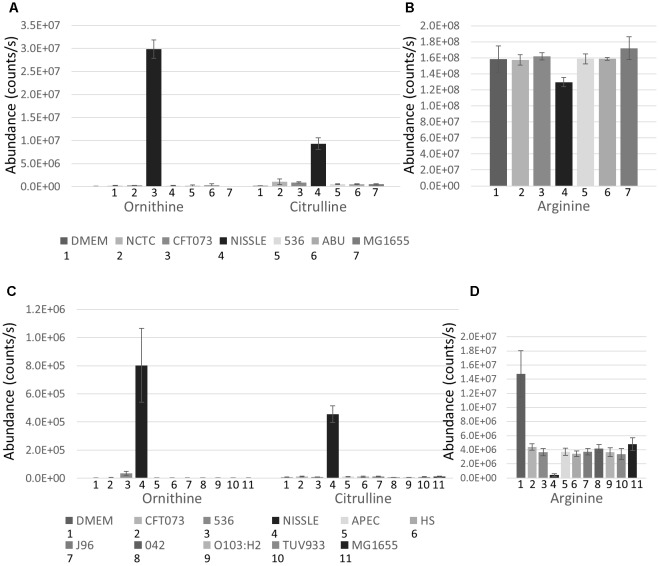
B2 focussed extracellular abundance of Arginine biosynthesis metabolites: **(A)** Average levels (counts/s) of Ornithine and Citrulline in DMEM medium and supernatant of 6 *E. coli* strains including 5 from phylogroup B2 and the laboratory reference strain MG1655 determined from 5 replicates. Nissle releases substantially higher levels of Ornithine and Citrulline into its environment than the other *E. coli* strains measured. **(B)** Level (counts/s) of Arginine in DMEM media and supernatant of the 6 *E. coli* strains. Nissle was found to deplete more Arginine from the media than the other strains measured. Extracellular abundance of Arginine biosynthesis metabolites identified in Study 1: **(C)** Average levels (counts/s) of Ornithine and Citrulline in DMEM medium and supernatant of 10 *E. coli* strains including 5 from phylogroup B2 and 5 from other phylogroups. Nissle secretes both Ornithine and Citrulline in much higher quantities than the other 9 strains measured. **(D)** Average levels (counts/s) of Arginine in DMEM media and supernatant of the 10 *E. coli* strains. Nissle was found to deplete more Arginine from the media than the other 9 strains analyzed.

The strains used for detailed B2 metabolome analysis are phylogenetically closely related and have been demonstrated to share phenotypic and pathogenicity determinants (Sun et al., 2005; Hancock et al., 2010; Vejborg et al., 2010; summary table presented as Supplementary Table S2). Nonetheless, these strains display different phenotypes ranging from pathogenic to probiotic, resulting at least in part from variation in genome island content (Sun et al., 2005; Hancock et al., 2010; Vejborg et al., 2010). Supernatants of five B2 strains complemented with the widely used MG1655 strain were analyzed using hydrophilic liquid chromatography (pHILIC) coupled to mass spectrometry (MS) and MS fragmentation (MS/MS) analyses. This comparative metabolomics profiling showed clear inter-strain differences. Notably, the B2 phylogroup strains revealed several identified metabolites (matching to standards run under same conditions with the samples) to be present in the supernatant at significantly different levels despite their close phylogenetic relatedness. Amongst those metabolites, Ornithine and Citrulline (see Supplementary Figure S1 for all structures discussed in this paper) were amongst the most substantially differential metabolites with all exhibiting higher levels in the supernatant of the Nissle strain. Comparison of Ornithine and Citrulline levels across all strains (and the DMEM medium control) indicates that Nissle is clearly distinct in its metabolomic output (Figure 2A). Concomitantly, Arginine was more depleted from the media by Nissle than any other strain (Figure 2B). We then also compared the levels of these three metabolites in the first study (Figure 2C,D) which show consistent changes with the B2 focused study. Moreover, a B2 focused validation study (Study 3) was performed using samples from cultures prepared separately – again showing similar metabolic profiles for the Arginine biosynthesis pathway related metabolites (Supplementary Figure S2). Thus, we could verify across three independent experiments (Studies 1 to 3) that for the identified metabolites the observed changes between Nissle and other strains were consistent (Figure 2 and Supplementary Figure S2). Hence, differences in the Arginine biosynthesis pathway-related metabolites appear to be occurring in Nissle compared with the other B2 strains examined.

**FIGURE 3:**
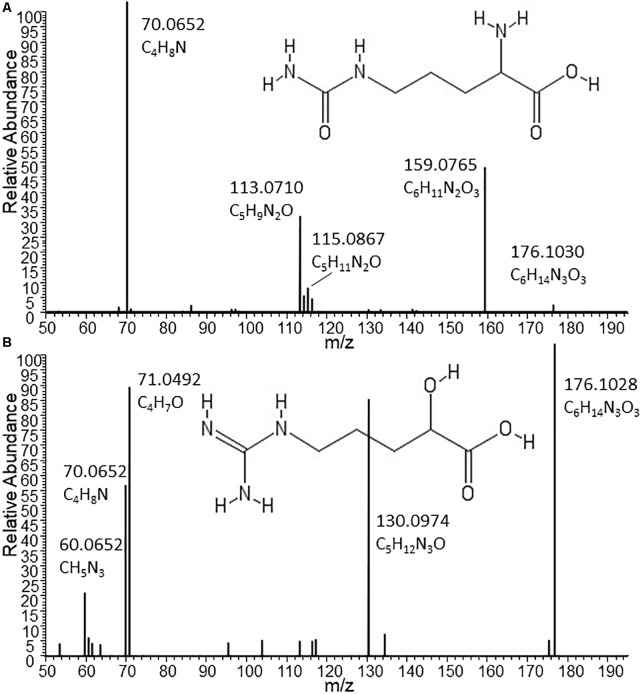
Structures and MS/MS spectra from Citrulline **(A)** and Arginic acid **(B)** as found in supernatants of Nissle and CFT073, respectively. The two isomers nearly co-elute under the chromatographic conditions used for this study. Only by MS/MS experiments it could be established that the C_6_H_13_N_3_O_3_ metabolite released by the phylogenetically closely related strains CFT073 (as well as NCTC12241/ATCC25922) can be annotated as Arginic acid. Spectrum **(A)** from Nissle could be matched to reference MS/MS spectra of Citrulline. Absent from **(A)**, the most intense fragment in spectrum B obtained from CFT073 is C_4_H_7_O, which indicates the likely presence of an C4-alkyl chain with a hydroxyl group (as present in Arginic acid); moreover, the CH_5_N_3_ fragment corresponding to the guanine group of Arginic acid is also present in **B** and absent from **A**.

Given the low but consistent levels of Citrulline detected in other B2 strains coupled with much lower (or lack of) Ornithine levels, we assessed LC-MS/MS spectra from the metabolites detected in Nissle and other strains (Figure 3). Indeed, with the MS/MS spectrum for the C_6_H_13_N_3_O_3_ metabolite obtained from Nissle we could confirm Citrulline’s structure (Figure 3A). However, the fragmentation spectra obtained for the C_6_H_13_N_3_O_3_ metabolite in other B2 strains such as CFT073 (Figure 3B) showed clear differences from Citrulline. With manual interpretation of the resulting MS/MS mass fragments, we annotated this metabolite as Arginic acid (a.k.a. Argininic acid). Indeed, a standard confirmed Arginic acid to elute 0.01 min from Citrulline (10.99 versus 11.00 min – well within 5% RT window to match peaks across different samples) and its differential fragmentation pattern from Citrulline. Thus, we observed differences in Arginine biosynthesis-related metabolites uniquely associated with Nissle.

### Substantial Differences in Arginine and Citrulline Related Metabolites in Nissle

**FIGURE 4:**
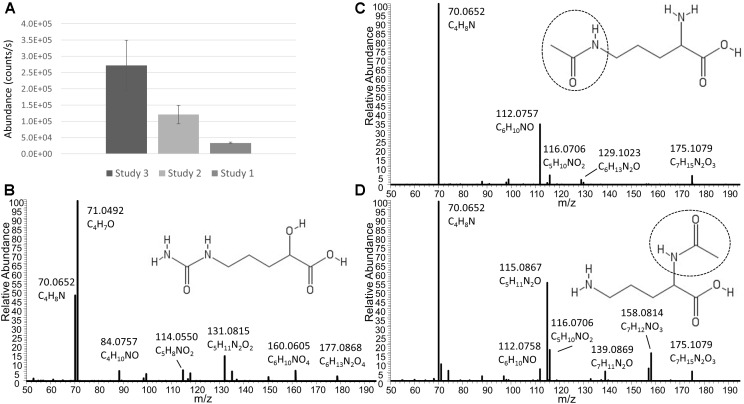
**(A)** Average levels (counts/s) of a metabolite released only by Nissle in three independent experiments (Studies 1, 2, and 3). Note that the absolute levels are dependent on many factors such as column bleeding, matrix effects, co-elution of metabolites, amongst others. The metabolite was not detected in any other strain investigated. The elemental formula of C_6_H_12_N_2_O_4_ and its fragmentation spectrum shown in **(B)** indicate its relatness to Citrulline and Arginic acid. Via use of MAGMa (see Supplementary Figure S3) and manual inspection of the MS/MS spectrum, the structural annotation of 5-(Carbamoylamino)-2-hydroxypentanoic acid was established. Panel **(C)** shows the Nissle LC-MS/MS spectrum of N5-acetylornithine which was released by Nissle, CFT073, and NCTC12241/ATCC25922. Note the absence of the NH_3_ loss in this spectrum. Other strains in the study released N2-acetylornithine (spectrum shown in **(D)** obtained from MG1655) or no acetylornithine at all. Note the presence of the NH_3_ loss, indicative for the presence of an amine group not directly next to a carboxylic acid group. Spectrum D also matched to reference spectra in the Metlin database. There was 0.4 min RT difference between the two N-acetylornithine isomers in the gradient used – with the N5 isomer eluting earlier.

**FIGURE 5:**
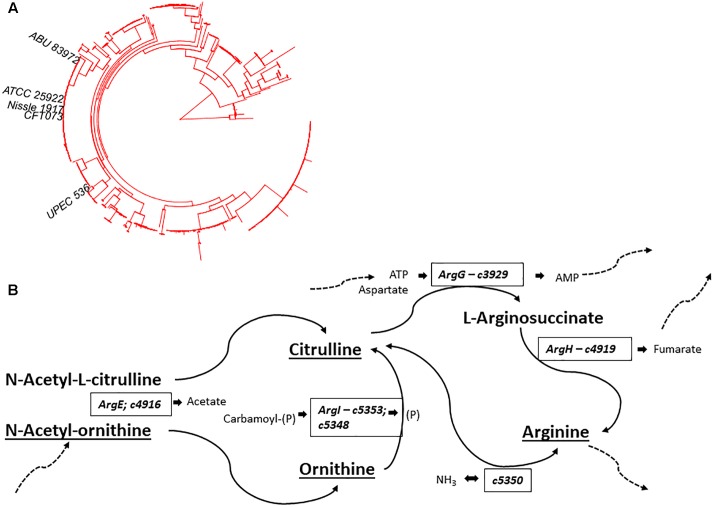
**(A)** Phylogenetic position of B2 strains in Studies 2 and 3 (B2-focussed and validation studies) on the B2 phylogroup phylogenetic tree – notice the thigh grouping of NCTC12241/ATCC25922, Nissle 1917, and CFT073. **(B)** Schematic overview of Arginine biosynthesis relevant gene products with *E. coli* CFT073 as model. Compounds identified and annotated in this study are indicated by underlining. Additional substrates and products are indicated in small lettering adjacent to relevant enzymes. Dashed arrows indicate linkage to additional metabolic pathways. Comparative analysis showed identical Arginine deimidase (c5350 - enzyme EC 3.5.3.6) to be present in B2 strains Nissle, CFT073 and NCTC12241/ATCC25922. Identical amino acid sequences were also observed across these strains for enzymes EC 6.3.4.5 (ArgG – c3929), EC 4.3.2.1 (ArgH – c4919), EC 2.1.3.3 (ArgI – c5353; c5348) & EC 3.5.1.16 (ArgE – c4916). Thus, *E. coli* strains Nissle 1971, ABU87932 and NCTC12241/ATCC25922 share all indicated gene products whilst *E. coli* MG1655 lacks an orthologue of c5350 to interconvert Arginine and Citrulline.

In addition to Ornithine and Citrulline, we observed a metabolite with the elemental formula of C_6_H_12_N_2_O_4_ to be consistently present only in Nissle supernatant (Figure 4A). An LC-MS/MS spectrum was obtained (Figure 4B) and with manual interpretation and the use of MAGMa (Ridder et al., 2013), we were able to structurally annotate the metabolite as 5-(Carbamoylamino)-2-hydroxypentanoic acid (i.e., Citrulline backbone with the NH_2_ group exchanged for an OH group). Furthermore, we observed that N-acetylornithine was amongst differential metabolites comparing Nissle to MG1655 that is widely used as reference strain. Interestingly, with use of LC-MS/MS, we established that Nissle, CFT073, and NCTC12441/ATCC25922 all released N5-acetylornithine, whereas other strains, either lacked this or released its more commonly found isomer N2-acetylornithine (Figure 4C,D). Figure 5 shows the phylogenetic relatedness of B2 strains subject to the focussed study (Figure 5A) and salient steps in the Arginine biosynthesis pathway (Figure 5B). The B2 strains subject to the focussed metabolome analyses all possess in common all Arginine biosynthesis genes; strain MG1655 lacks arginine deiminase (EC 3.5.3.6, c5350 in Figure 5) which directly inter-converts Arginine and Citrulline.

*In silico* comparison of protein sequences from Nissle against CFT073, NCTC12441/ATCC25922 and ABU_83972 (Supplementary Table S3) identified 65 proteins which are present in Nissle but absent or divergent in the others. Additionally, forty-eight proteins which are conserved in CFT073, NCTC12441/ATCC25922 and ABU_83972 show divergence in Nissle; of these, the majority (*n* = 36) of divergent proteins differed by 1 to 3 amino acid substitutions whilst the other 12 showed short in-frame deletion (CIW80_16080) or insertion (CIW80_09850) or more substantial truncation. Among these 113 proteins, only one (CIW80_01615) was ascribed to an Arginine-related annotation, specifically Arginine N-succinyltransferase.

## Discussion and Conclusion

Interaction of bacteria with their environments (including mammalian hosts) has evolved complex series of interplays. From the bacterial perspective, major focus has been towards surface structures [e.g., capsule polysaccharides (Hafez et al., 2010), lipopolysaccharides (Grozdanov et al., 2002), fimbriae (Kleta et al., 2014), flagella (Schlee et al., 2007)], exported macromolecules (including toxins, effectors and their secretion systems) and nutrient (e.g. iron and carbon source) acquisition systems, all of which substantially contribute to niche occupation. Although much less attention has been directed towards the characterization of small exported molecules, recent years have seen a rise in awareness of the potential significance of bacterial metabolites in the interplay between bacteria and their hosts (Donia and Fischbach, 2015) and metabolomics approaches were applied to link metabolic potential to uropathogenic *E. coli* virulence factors (AlRabiah et al., 2014).

Our study profiled extracellular metabolomes of closely- and more distantly-related *E. coli* strains as proxy of their small molecule interactions with their immediate environments, i.e., the host and co-colonizing bacteria. Focussing on closely-related *E. coli* strains identified subtle differences in metabolite profiles which may relate to adoption of specific lifestyles and potentially contribute to interactions of these phenotypically distinct strains with their host.

Preliminary metabolome analysis included ten phylogenetically diverse *E. coli* encompassing representatives of phylogroups A, B1, B2, D & E and comprising intestinal pathogenic, extra-intestinal pathogenic, commensal and probiotic strains (Supplementary Table S1). Comparisons of extracellular metabolomes indicated clear distinctions among *E. coli* strains although it was not a goal to assess the extent to which any differences corresponded with genotype as phylogroups were mostly represented by one or two strains. Nonetheless, our observation of strain-dependent metabolome provides an initial perspective on the as yet unexplored level in the metabolome diversity among *E. coli* strains and any relatedness to metabolic capabilities, phylogenetic position and, importantly, pathotype or other lifestyle trait.

*Escherichia coli* within phylogroup B2 includes intestinal commensal strains and some enteric pathogens (such as enteropathogenic strain E2348/69 and adherent-invasive strains (such as LF82) associated with Crohn’s Disease) but is much more typically associated with invasive or extra-intestinal pathogenicity. These extra-intestinal pathogenic *E. coli* (ExPEC) survive both within the intestinal tract and in other tissue sites causing infections such as cystitis (UPEC), neonatal meningitis (NMEC), sepsis (SEPEC) and pneumonia. These infections often have severe clinical outcomes requiring extensive antibiotic and supportive treatment, however these pathogenic classes within *E. coli* phylogroup B2 are closely related to strains causing asymptomatic infection [e.g., strain ABU83972; (Dobrindt et al., 2016)] as well as the probiotic strain Nissle 1917 that has been shown to show antagonistic effects on pathogenic *E. coli* strains (Kleta et al., 2014; Rund et al., 2013). *E. coli* strains CFT073, NCTC12241/ATCC25922, ABU83792 and Nissle are reported as genomically closely related and share many virulence-associated determinants (Supplementary Table S2; Dobrindt et al., 2010; Hancock et al., 2010; Vejborg et al., 2010). Previously, a comparative analysis of metabolic potential of *E. coli* (including CFT073 and Nissle strains) using *in silico* approaches did not identify any differences (Sun et al., 2005); however, metabolic activities were not directly examined. Thus, our subsequent metabolome analyses focussed on isolates within this closely-related cluster to use the resolving capabilities of pHILIC coupled to high-resolution MS-based metabolomics to assess whether any of the strains could be distinguished in respect of extracellular metabolome profiles.

The application of pHILIC based LC-MS metabolic profiling of supernatants of distinct yet related *E. coli* strains revealed substantial differences in their extracellular metabolic profiles – verified in three independent analyses –, notably in metabolites associated with Arginine biosynthesis (many of which form part of the urea cycle in ureotelic organisms). Whilst B2 strains included in the focussed study possess Arginine deiminase (and other Arginine biosynthesis enzymes), only Nissle showed increased levels of Ornithine and Citrulline as well as a structurally related novel bacterial metabolite 5-(Carbamoylamino)-2-hydroxypentanoic acid in supernatant. These compounds differ through presence and/or position of ketone and amide groups and, although it is tempting to speculate that these may represent heretofore unrecognized intermediaries, signaling molecules, or bioactives, any mechanism through which these small molecular differences are established remains to be elucidated.

A comparison of protein-coding sequences from CFT073, NCTC12241/ATCC25922 and ABU_83972 identified 113 sequences which were conserved in these strains but divergent in Nissle (Supplementary Table S3). Of these, only one protein was annotated with function related to an Arginine-related metabolic process; this enzyme – Arginine-N-succinyltransferase – catalyzes the reaction succinyl-CoA + L-Arginine ⇌ CoA + N2-succinyl-L-Arginine, hence appears unlinked to metabolites observed in Nissle but not the others. As noted above (Figure 5B), there are no evident polymorphisms in sequences of the Arginine biosynthesis-related enzymes that may account for differences observed. Furthermore, it is not evident that functional categorization of gene products from operons co-ordinately regulated with Arginine biosynthesis loci provide the necessary enzymatic activities to generate either 5-(Carbamoylamino)-2-hydroxypentanoic acid or Arginic acid; however, it is conceivable that gene products currently annotated as “hypothetical” may confer relevant biosynthetic capacities.

Coupled with metabolomics, extensive, systematic interrogation of genes, operons and regulons in Nissle and closely-related strain would be required to define which gene products were involved in the observed divergence in metabolomes among these strains. The influence of availability of particular nutrients on metabolome is also a pertinent aspect. For example, our own investigations with *Campylobacter jejuni* (van der Hooft et al., 2018) have shown that supplementation of defined medium with single components (glutamate or fucose) can substantively affect extracellular metabolome composition. Hence, it is conceivable that alteration of nutrient availability may affect metabolome of *E. coli* strains and this issue will be important to pursue in future investigations as this might enable alteration in pathotype or even improvement in probiotic functionality. This investigation presents multiple hypotheses for further pursuit, notably the extent to which *E. coli* strains produce similarly divergent metabolome profiles and comprehension of association with pathotypes remains to be assessed.

The annotation of 5-(Carbamoylamino)-2-hydroxypentanoic acid and the two N-Acetylornithine isomers as well as the identification of Arginic acid do question whether the Arginine biosynthesis pathway is completely characterized and further characterization would be required to define the enzymes contributing to synthesis of these “orphan” metabolites and, potentially, their roles in physiology of these *E. coli* strains. With complete genomes now available for an increasing amount of bacterial strains including Nissle (Cress et al., 2013; Reister et al., 2014), more insight in biosynthesis pathways and responsible genes will hopefully accrue.

The novel insight into metabolic capacities of closely related *E. coli* strains through high resolution metabolomics has identified subtle differences in compound identities despite presence of ostensibly identical Arginine biosynthetic pathways in these strains. The specification of structurally distinct isomeric compounds among these strains gives cause to postulate that these may influence bacterial fitness and interactions within their host. For instance, production and release of metabolites such as indole-based metabolites or nucleotides by bacteria have been linked to cellular, tissue, and host well-being (Idzko et al., 2014; Berstad et al., 2015). Similarly, there are several studies indicating that citrulline may have beneficial effects to the host (Chapman et al., 2012; Bahri et al., 2013; Breuillard et al., 2015; Antunes et al., 2016; Lai et al., 2017). In contrast, a study found that Arginic Acid promotes oxidative damage (Delwing-de Lima et al., 2017) and the molecule was found among a panel of metabolites associated with chronic kidney disease (Zhang et al., 2016). It is also noteworthy that Arginine metabolism has been associated earlier to pathogenicity during an investigation of the impact of expression of yersiniabactin-related genes on the primary metabolism of *E. coli* (Lv and Henderson, 2011), an iron uptake system present in B2 strains subject to our focussed metabolome analysis. The potential biological significance of single exported metabolites cannot be overlooked - a recent report (Alvarez et al., 2017) identified that D-arginine released by the enteric pathogen *Vibrio cholerae* results in modulation of enteric microbiota. Whether Arginic acid, Citrulline, Ornithine or other extracellular metabolites have comparable bioactivities will be subject to confirmation through further investigation.

The observation that only Nissle produced Citrulline whereas pathogenic relatives produced Arginic acid, shows unexpected correspondence to the pathotypes of the strains. Any causal relationship between compound synthesis and release with outcome during interaction with co-colonizers or the host has yet to be established; however, it is clear from this metabolomic study that the individual strains show remarkable unexpected differences in their metabolic capacities. Further determination will (i) require detailed understanding of *E. coli* determinants responsible for synthesis of these compounds, combined with structural confirmation of compounds produced, (ii) investigation of the production of arginine biosynthesis-related metabolites in more *in vivo*-like settings, and (iii) establishing the potential influence of Ornithine and Arginine on the host and co-colonizers of the gut (the gut microbiota).

In summary, detailed and accurate metabolomic profiling such as that in this study highlights how deepening characterization of metabolic capabilities may make a significant contribution to understanding bioactivities of organisms. This metabolomics approach has offered novel and intriguing insights into determinants of functional differences between genetically closely related *E. coli* strains which warrant further investigation into the contribution of metabolites to probiotic and pathogenic characteristics. Just after the centenary year of *E. coli* Nissle 1917 we establish further its distinctiveness from related strains in extracellular metabolome and reveal aspects that may contribute to its potent probiotic characteristics and which are worthy of further evaluation.

## Author Contributions

JH and DS conceived and designed the work. SH and DS generated the sample materials. Mass spectrometry measurements were performed at Glasgow Polyomics supervised by KB. JH carried out mass spectrometry fragmentation experiments and processed and analyzed the resulting metabolomics data. JH, RG, and DS analyzed and interpreted the data and composed the manuscript. All authors contributed to further appraisal, editing of the work, and approved content for submission.

## Conflict of Interest Statement

The authors declare that the research was conducted in the absence of any commercial or financial relationships that could be construed as a potential conflict of interest.

## Footnotes

1 http://polyomics.mvls.gla.ac.uk

2 www.mzcloud.org

3 metlin.scripps.edu

4 www.emetabolomics.org
