# Supplementary material for: Substantial Extracellular Metabolic Differences Found Between Phylogenetically Closely Related Probiotic and Pathogenic Strains of Escherichia coli
Source: Front Microbiol. 2019 Feb 19;10:252. doi: 10.3389/fmicb.2019.00252 (PMC6390828; doi:10.3389/fmicb.2019.00252)
Supplement: Supplementary file 1 [file Data_Sheet_1.docx]

**Supplementary Information to:**

**Substantial extracellular metabolic differences found between phylogenetically closely related probiotic and pathogenic strains of *Escherichia coli***

Justin J J van der Hooft^1*^

Robert J Goldstone^2^

Susan Harris^2^

Karl E V Burgess^1^

David G E Smith^2*^

^1^ Glasgow Polyomics, College of Medical, Veterinary and Life Sciences, University of Glasgow, Glasgow G12 8QQ.

^2^ Institute of Biological Chemistry, Biophysics & Bioengineering, Heriot-Watt University, Edinburgh, EH14 4AS.

^*^ corresponding authors

**SUPPLEMENTARY TABLE S1. Strains used in this study**

| **Strain** | **PG** | **Description** | **Assembly designation** |
| --- | --- | --- | --- |
| UPEC 536 | B2 | Wild-type urinary pathogenic *E. coli* | GCA_000013305.1 |
| APEC O1 | B2 | Wild-type avian pathogenic *E. coli* | GCA_000014845.1 |
| CFT073 | B2 | Wild-type urinary pathogenic *E. coli* | GCA_000007445.1 |
| J96 | B2 | Wild-type urinary pathogenic *E. coli* | GCA_000295775.2 |
| Nissle 1917 | B2 | Probiotic *E. coli* strain | GCA_003546975.1 |
| NCTC12241 /  ATCC25922 | B2 | Wild-type clinical isolate | GCA_000743255.1 |
| ABU83972 | B2 | Asymptomatic bacteriuria isolate | GCA_000148365.1 |
| 042 | D | Wild-type Enteroaggregative *E. coli* strain | GCA_000027125.1 |
| MG1655 | A | *E. coli* K-12 type strain | GCA_000005845.2 |
| HS | A | Wild-type commensal *E. coli* | GCA_000017765.1 |
| O103:H2 | B1 | Wild-type intestinal pathogenic *E. coli* | No genome information |
| TUV93-0 | E | Natural *stx-*phage deletion of EDL933, enterohaemorrhagic *E. coli* | GCA_000006665.1  (EDL933 genome) |


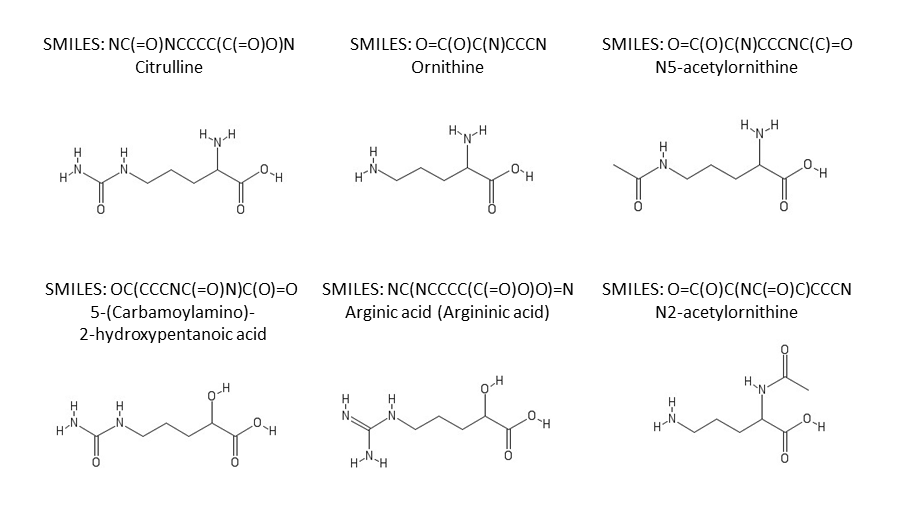
**SUPPLEMENTARY FIGURE S1** Structures of 6 Arginine and Citrulline related metabolites discussed in this study.


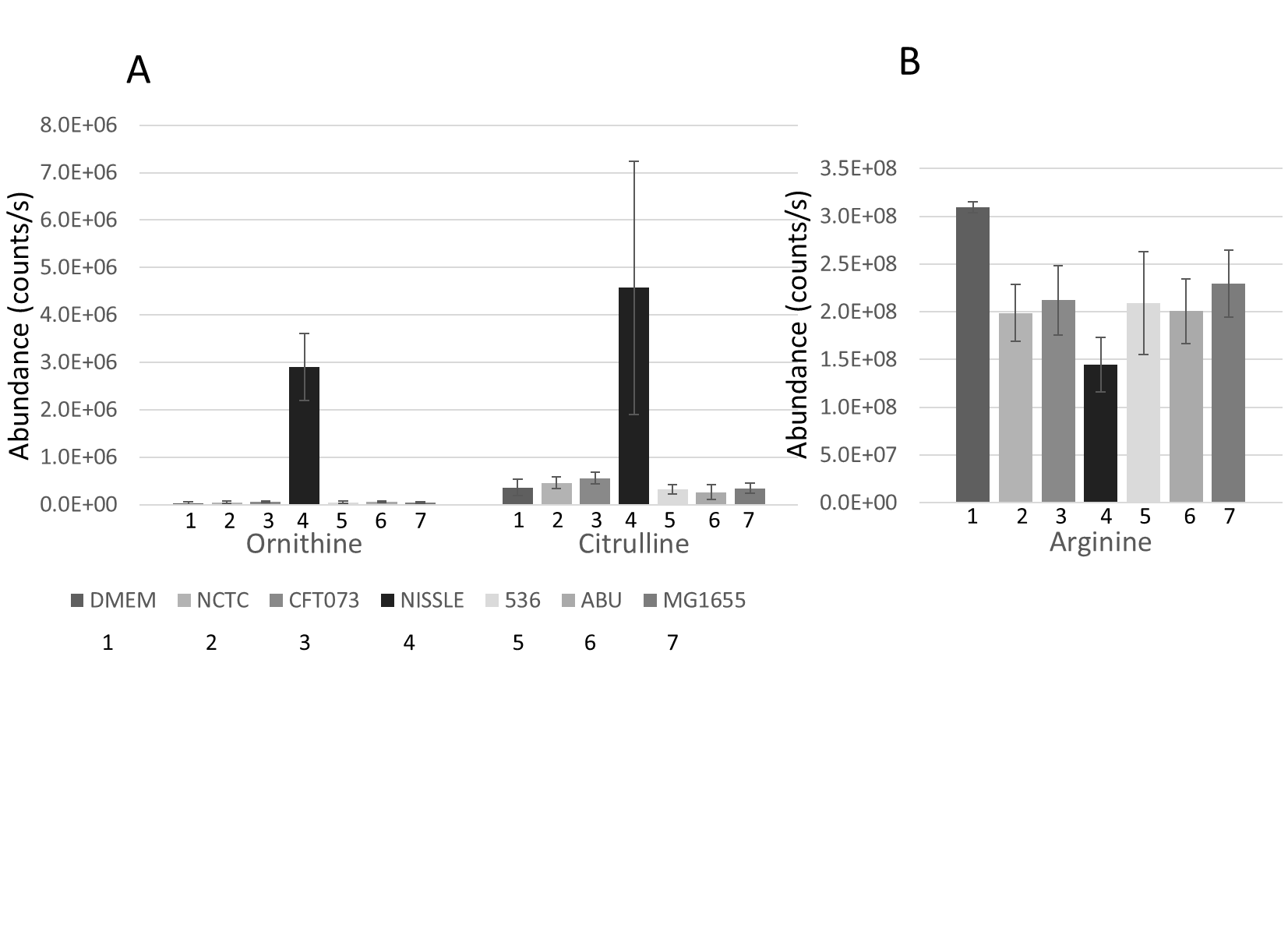


**SUPPLEMENTARY FIGURE S2** – B2 focused validation study **A**) Average levels (counts/s) of Ornithine and Citrulline in DMEM medium and supernatant of 6 *E. coli* strains including 5 from phylogroup B2 and the laboratory reference strain MG1655 determined from 5 biological replicates of Study 2. Nissle releases substantially higher levels of Ornithine and Citrulline into its environment than the other *E. coli* strains measured. Note that the Ornithine levels in A are from negative ionization mode as the positive ionization mode LC-MS peak was not peak picked by the software – manual inspection revealed average levels of 1.6E7 in positive ionization mode. **B**) Level (counts/s) of Arginine in DMEM media and supernatant of the 6 *E. coli* strains. Nissle was found to deplete more Arginine from the media than the other strains measured. The B2 focused validation experiment showed the changes in Arginine biosynthesis pathway metabolites to be consistent.


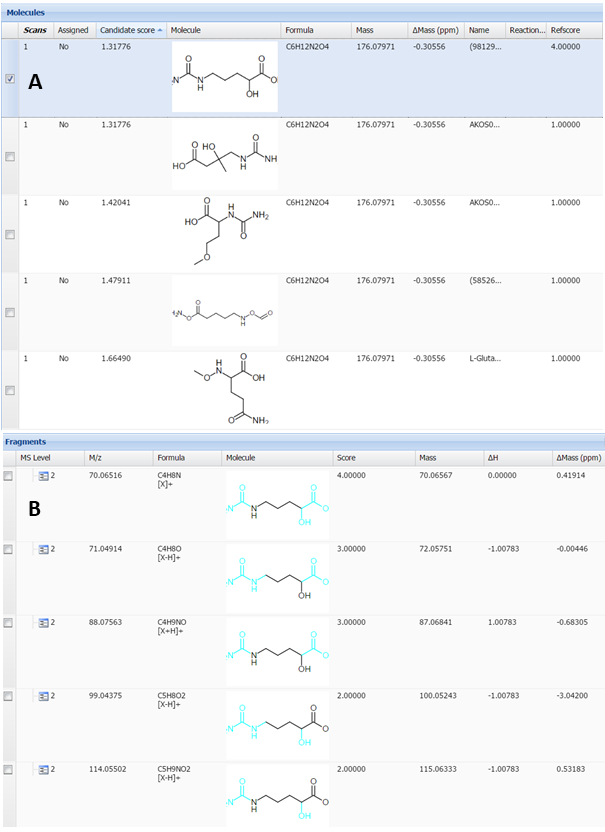


**SUPPLEMENTARY FIGURE S3** Structural annotation of 5-(Carbamoylamino)-2-hydroxypentanoic acid with aid of MAGMa. In Pubchem, 445 candidates were found within 5 ppm of the precursor mass, of which 277 were C6H12N2O4 isomers. **A**) shows the top 5 ranked candidates based on (lowest) candidate scores. **B**) shows how fragments found in the MS/MS spectrum could be explained mapped on the 5-(Carbamoylamino)-2-hydroxypentanoic acid structure, where parts in black represent the mass fragments. Both C_4_H_8_O and C_4_H_8_N fragments can be readily explained in this candidate structure.

**SUPPLEMENTARY TABLE S2 – Distribution of fitness-, colonisation- and virulence-associated factors of *Escherichia coli* CFT073 among closely-related strains used in this study.**

| **Locus** | **Description** | **CFT073** | **Nissle** | **ATCC25922** | **ABU83972** | **536** | **reference** |
| --- | --- | --- | --- | --- | --- | --- | --- |
| *iucABCD_iutA* | Aerobactin | C3623-c3627 |  |  |  |  | Torres et al., 2001 |
| *IroBCDEN* | Salmochelin | C1250-c1254 |  |  |  |  | Porcheron et al., 2014 |
| *ybtSXQPA_irp2_irp1_ybtUTE_fyuA* | Yersiniabactin | C2426-c2436 |  |  |  |  | Garcia et al., 2011 |
| *fbpABCD* | Iron acquisition | C0294-c0297 |  |  |  |  | Lloyd et al., 2009a |
| *chuSA* | Haem acquisition/utilisation | C4307-c4308 |  |  |  |  | Smith et al., 2010 |
| *Hma* | Haem acquisition | C2482 |  |  |  |  | Hagan & Mobley. 2009 |
| *znuABC* | Zinc uptake | C2271-c2273 |  |  |  |  | Sabri et al., 2009 |
| *dsdCXA* | D-serine uptake and catabolism | c2899-c2901 |  |  |  |  | Hryckowian et al., 2015 |
| *oppA* | Peptide transporter | C1707 |  |  |  |  | Subashchandrabose et al., 2013 |
| *Kgu* | α-ketoglutarate metabolism island | c5032-c5041 |  |  |  |  | Cai et al., 2013 |
| *pgaABCD* | Poly-N-acetyl-glucosamine biosynthesis | C1160-c1164 |  |  |  |  | Subashchandrabose et al., 2013 |
| *neaT* | Putative acetyltransferase | C0970 |  |  |  |  | Wiles et al., 2013 |
| *pafRP* | Sensor-regulator | c3408-c3409 |  |  |  |  | Baum et al., 2014 |
| *hlyCABD* | Haemolysin | c3569-c3570-c3573-c3574 |  |  |  |  | Kao et al., 1997 |
| *sapABCDF* | Sensitivity to antimicrobial peptides | C1767-c1771 |  |  |  |  | Subashchandrabose et al., 2013 |
| *tosA* | RTX exotoxin | C0363 |  |  |  |  | Lloyd et al., 2009a |
| *sisA* | immunomodulator | C3557 |  |  |  |  | Lloyd et al. 2009b |
| *sisB* | Immunomodulator | C4492 |  |  |  |  | Lloyd et al. 2009b |
| *tcpC* | TIR domain protein | C2398 |  |  |  |  | Yadav et al., 2010 |
| *pic* | Serine protease/mucinase | C0350 |  |  |  |  | Subashchandrabose et al., 2013 |
| *vat* | Vacuolating autotransporter toxin | C0393 |  |  |  |  | Subashchandrabose et al., 2013 |
| *sat* | Secreted autotransporter toxin | C3619 |  |  |  |  | Guyer et al., 2002 |
| *ag43a* | Autotransporter adhesin | c3655 |  |  |  |  | Ulett et al., 2007 |
| *upaB* | Autotransporter adhesin | c0426 |  |  |  |  | Allsop et al., 2012 |
| *upaH/ ydbA_1-ydbA_2* | Autotransporter adhesin | c5621_c1831 |  |  |  |  | Allsop et al., 2010 |
| *esiB* | sIgA binding protein | c5321 |  |  |  |  | Pastorello et al., 2013 |
| *Iha* | iron-regulated gene homologue adhesin | c3610 |  |  |  |  | Johnson et al., 2005 |
| *pap_1* | P-fimbria | c3582-c3593 |  |  |  |  | Mobley et al., 1993 |
| *pap_2* | P-fimbria | c5178-c5189 |  |  |  |  | Mobley et al., 1993 |
| *ygiLGH* | Fimbrial adhesin | c3791-c3793 |  |  |  |  | Spurbeck et al., 2011 |
| *c2394-c2395* | Type IV pilus | c2394-c2395 |  |  |  |  | Subashchandrabose et al., 2013 |

Footnote: black shading indicates a protein sequence with ≥90% identity; grey shading indicates a match at 60-90% identity or are possible pseudogenes.

**SUPPLEMENTARY REFERENCES SUPPLEMENTARY TABLE S2**

Allsopp LP, Totsika M, Tree JJ, Ulett GC, Mabbett AN, Wells TJ, Kobe B, Beatson SA, Schembri MA. (2010). UpaH is a newly identified autotransporter protein that contributes to biofilm formation and bladder colonization by uropathogenic *Escherichia coli* CFT073. *Infection and Immunity* **78**(4):1659-1669.

Allsopp LP, Beloin C, Ulett GC, Valle J, Totsika M, Sherlock O, Ghigo JM, Schembri MA. (2012). Molecular characterization of UpaB and UpaC, two new autotransporter proteins of uropathogenic *Escherichia coli* CFT073. *Infection and Immunity* **80**(1):321-332.

Baum M, Watad M, Smith SN, Alteri CJ, Gordon N, Rosenshine I, Mobley HL, Amster-Choder O. (2014). PafR, a novel transcription regulator, is important for pathogenesis in uropathogenic *Escherichia coli*. *Infection and Immunity* **82**(10):4241-4252.

Cai W, Wannemuehler Y, Dell'anna G, Nicholson B, Barbieri NL, Kariyawasam S, Feng Y, Logue CM, Nolan LK, Li G. (2013). A novel two-component signaling system facilitates uropathogenic *Escherichia coli*'s ability to exploit abundant host metabolites. *PLoS Pathogens* **9**(6):e1003428.

Garcia EC, Brumbaugh AR, Mobley HL. (2011). Redundancy and specificity of *Escherichia coli* iron acquisition systems during urinary tract infection. *Infection and Immunity* **79**(3):1225-1235.

Guyer DM, Radulovic S, Jones FE, Mobley HL. (2002). Sat, the secreted autotransporter toxin of uropathogenic *Escherichia coli*, is a vacuolating cytotoxin for bladder and kidney epithelial cells. *Infection and Immunity* **70**(8):4539-4546.

Hryckowian AJ, Baisa GA, Schwartz KJ, Welch RA. (2015). *dsdA* Does Not Affect Colonization of the Murine Urinary Tract by *Escherichia coli* CFT073. *PLoS One* **14**;10(9):e0138121.

Johnson JR, Jelacic S, Schoening LM, Clabots C, Shaikh N, Mobley HL, Tarr PI. (2005). The IrgA homologue adhesin Iha is an *Escherichia coli* virulence factor in murine urinary tract infection. *Infection and Immunity* **73**(2):965-971.

Kao JS, Stucker DM, Warren JW, Mobley HL. (1997). Pathogenicity island sequences of pyelonephritogenic *Escherichia coli* CFT073 are associated with virulent uropathogenic strains. Infection and Immunity **65**(7):2812-2820.

Lloyd AL, Henderson TA, Vigil PD, Mobley HL. (2009a). Genomic islands of uropathogenic *Escherichia coli* contribute to virulence. *Journal of Bacteriology* **191**(11):3469-3481.

Lloyd AL, Smith SN, Eaton KA, Mobley HL. (2009b). Uropathogenic *Escherichia coli* Suppresses the host inflammatory response via pathogenicity island genes *sisA* and *sisB*. *Infection and Immunity* **77**(12):5322-5333.

Pastorello I, Rossi Paccani S, Rosini R, Mattera R, Ferrer Navarro M, Urosev D, Nesta B, Lo Surdo P, Del Vecchio M, Rippa V, Bertoldi I, Gomes Moriel D, Laarman AJ, van Strijp JA, Daura X, Pizza M, Serino L, Soriani M. (2013). EsiB, a novel pathogenic *Escherichia coli* secretory immunoglobulin A-binding protein impairing neutrophil activation. *mBio* **4**(4). pii: e00206-13.

Porcheron G, Habib R, Houle S, Caza M, Lépine F, Daigle F, Massé E, Dozois CM. (2014). The small RNA RyhB contributes to siderophore production and virulence of uropathogenic *Escherichia coli*. *Infection and Immunity* **82**(12):5056-5068.

Sabri M, Houle S, Dozois CM. (2009). Roles of the extraintestinal pathogenic *Escherichia coli* ZnuACB and ZupT zinc transporters during urinary tract infection. *Infection and Immunity* **77**(3):1155-1164.

Smith SN, Hagan EC, Lane MC, Mobley HL. (2010). Dissemination and systemic colonization of uropathogenic *Escherichia coli* in a murine model of bacteremia. *mBio* **1**(5). pii: e00262-10.

Spurbeck RR, Stapleton AE, Johnson JR, Walk ST, Hooton TM, Mobley HL. (2011). Fimbrial profiles predict virulence of uropathogenic *Escherichia coli* strains: contribution of *ygi* and *yad* fimbriae. *Infection and Immunity* **79**(12):4753-4763.

Subashchandrabose S, Smith SN, Spurbeck RR, Kole MM, Mobley HL. (2013). Genome-wide detection of fitness genes in uropathogenic *Escherichia coli* during systemic infection. *PLoS Pathogens* **9**(12):e1003788.

Torres AG, Redford P, Welch RA, Payne SM. (2001). TonB-dependent systems of uropathogenic *Escherichia coli*: aerobactin and heme transport and TonB are required for virulence in the mouse. *Infection and Immunity* **69**(10):6179-6185.

Ulett GC, Valle J, Beloin C, Sherlock O, Ghigo JM, Schembri MA. (2007). Functional analysis of antigen 43 in uropathogenic *Escherichia coli* reveals a role in long-term persistence in the urinary tract. *Infection and Immunity* **75**(7):3233-3244.

Wiles TJ, Norton JP, Smith SN, Lewis AJ, Mobley HL, Casjens SR, Mulvey MA. (2013). A phyletically rare gene promotes the niche-specific fitness of an *E. coli* pathogen during bacteremia. PLoS Pathogens **9**(2):e1003175.

Yadav M, Zhang J, Fischer H, Huang W, Lutay N, Cirl C, Lum J, Miethke T, Svanborg C. (2010). Inhibition of TIR domain signaling by TcpC: MyD88-dependent and independent effects on *Escherichia coli* virulence. *PLoS Pathogens* **6**(9):e1001120.

**SUPPLEMENTARY TABLES S3A-C:**

**Summary of chromosome-encoded protein sequence divergence between *E. coli* strain Nissle 1917 and strains CFT073, NCTC12241 and ABU_83972.**

**SUPPLEMENTARY TABLE S3A – protein coding sequences present in *E. coli* strain Nissle 1917 and absent in strains CFT073, NCTC12241 and ABU_83972.**

| **Nissle locus tag** | **Match** | **Annotation** |
| --- | --- | --- |
| CIW80_02265 | none | hypothetical protein |
| CIW80_02270 | none | DNA polymerase V |
| CIW80_02275 | none | DinI family protein |
| CIW80_02280 | none | hypothetical protein |
| CIW80_02285 | none | tail fiber assembly protein |
| CIW80_02290 | none | tail assembly chaperone |
| CIW80_02295 | none | phage tail protein |
| CIW80_02300 | none | hypothetical protein |
| CIW80_02305 | none | host specificity protein |
| CIW80_02310 | none | tail assembly protein |
| CIW80_02315 | none | peptidase P60 |
| CIW80_02320 | none | phage minor tail protein L |
| CIW80_02325 | none | phage tail protein |
| CIW80_02330 | none | phage tail tape measure protein |
| CIW80_02335 | none | phage tail assembly protein T |
| CIW80_02340 | none | phage minor tail protein G |
| CIW80_02345 | none | phage tail protein |
| CIW80_02350 | none | phage tail protein |
| CIW80_02355 | none | phage tail protein |
| CIW80_02360 | none | DNA breaking-rejoining protein |
| CIW80_02365 | none | DUF2190 domain-containing protein |
| CIW80_02370 | none | peptidase S14 |
| CIW80_02375 | none | phage portal protein |
| CIW80_02380 | none | hypothetical protein |
| CIW80_02385 | none | DNA packaging protein |
| CIW80_02390 | none | DUF1441 domain-containing protein |
| CIW80_02395 | none | Fur-regulated protein |
| CIW80_02400 | none | Fur-regulated protein |
| CIW80_02405 | none | hypothetical protein |
| CIW80_02410 | none | hypothetical protein |
| CIW80_02415 | none | DUF2514 domain-containing protein |
| CIW80_02420 | none | lysozyme |
| CIW80_02425 | none | holin |
| CIW80_02430 | none | site-specific DNA-methyltransferase |
| CIW80_02435 | none | TrmB family transcriptional regulator |
| CIW80_02440 | none | serine protease |
| CIW80_02445 | none | hypothetical protein |
| CIW80_02450 | none | antitermination protein |
| CIW80_02455 | none | hypothetical protein |
| CIW80_02460 | none | phage regulatory protein/antirepressor Ant |
| CIW80_02465 | none | RusA family crossover junction endodeoxyribonuclease |
| CIW80_02470 | none | phage N-6-adenine-methyltransferase |
| CIW80_02475 | none | GntR family transcriptional regulator |
| CIW80_02480 | none | DUF4222 domain-containing protein |
| CIW80_02485 | none | hypothetical protein |
| CIW80_02490 | none | hypothetical protein |
| CIW80_02495 | none | XRE family transcriptional regulator |
| CIW80_02500 | none | Clp protease |
| CIW80_02505 | none | recombination-associated protein RdgC |
| CIW80_02510 | none | hypothetical protein |
| CIW80_02515 | none | hypothetical protein |
| CIW80_02520 | none | hypothetical protein |
| CIW80_02525 | none | hypothetical protein |
| CIW80_02530 | none | DUF550 domain-containing protein |
| CIW80_02535 | none | DUF4222 domain-containing protein |
| CIW80_02540 | none | 3'-5' exoribonuclease |
| CIW80_02545 | none | excisionase |
| CIW80_02550 | none | integrase |
| CIW80_08645 | none | IS1 family transposase |
| CIW80_08650 | none | pilus assembly protein |
| CIW80_08655 | none | transcriptional regulator |
| CIW80_13070 | none | transcriptional regulator |
| CIW80_14435 | none | ISL3 family transposase ISEc53 |
| CIW80_16750 | none | IS3 family transposase |
| CIW80_17180 | none | ISL3 family transposase ISEc53 |

**SUPPLEMENTARY TABLE S3B – protein coding sequences conserved in *E. coli* strains CFT073, NCTC12241 and ABU_83972 and truncated in strain Nissle 1917.**

| **Nissle locus tag** | **Matching CFT073 locus tag** | **% identity** | **Annotation** |
| --- | --- | --- | --- |
| CIW80_01615 | c2147 | 99.4 | arginine N-succinyltransferase |
| CIW80_02930 | c2354 | 99.6 | Flagellar M-ring protein |
| CIW80_02990 | c2367 | 97.3 | Flagellar biosynthetic protein FliR |
| CIW80_04055 | c2579 | 99.7 | GDP-mannose 4,6-dehydratase |
| CIW80_07200 | c3260 | 99.2 | Glucitol operon activator protein |
| CIW80_07245 | c3271 | 98.6 | Hypothetical protein |
| CIW80_08065 | c3447 | 99.8 | Hypothetical sigma-54-dependent transcriptional regulator YgeV |
| CIW80_08400 | c3512 | 89.3 | transcriptional regulator |
| CIW80_08925 | c3643 | 99.7 | Unknown in ISEc8 |
| CIW80_09590 | c3774 | 99.6 | Ferric enterobactin transport ATP-binding protein FepC |
| CIW80_11455 | c4159 | 99.7 | 3-dehydroquinate synthase |
| CIW80_11755 | c4222 | 99.5 | Putative DNA processing protein |
| CIW80_12015 | c4279 | 99.8 | PTS system, galactitol-specific IIC component |
| CIW80_12625 | c4403 | 99.8 | Cryptic L-xylulose kinase |
| CIW80_12665 | c4412 | 99.8 | L-seryl-tRNA(Sec) selenium transferase |
| CIW80_13290 | c4606 | 99.3 | 16 kDa heat shock protein B |
| CIW80_14210 | c4793 | 99.9 | Fatty oxidation complex alpha subunit |
| CIW80_14220 | c4795 | 99.7 | GNAT family N-acetyltransferase |
| CIW80_14420 | c4835 | 99.7 | Hypothetical protein YiiD |
| CIW80_15590 | c5064 | 99.8 | Acetyl-coenzyme A synthetase |
| CIW80_16525 | c5331 | 99.8 | UDP-N-acetylmuramate:L-alanyl-gamma-D-glutamyl-meso-diaminopimelate ligase |
| CIW80_17310 | c5414 | 98 | MFS transporter |
| CIW80_17695 | c0005 | 99.8 | Threonine synthase |
| CIW80_18170 | c0110 | 99.7 | D-alanine--D-alanine ligase |
| CIW80_18525 | c0187 | 99.7 | Ferrichrome-binding periplasmic protein precursor |
| CIW80_18950 | c0278 | 99.6 | Hypothetical protein YafZ |
| CIW80_19035 | c0296 | 99.7 | Hypothetical protein |
| CIW80_19035 | c2516 | 99.7 | ABC transporter, FecCD transport family |
| CIW80_20575 | c0604 | 99.9 | Copper-transporting P-type ATPase |
| CIW80_21255 | c0738 | 99.6 | Glutamate/aspartate transport system permease protein GltJ |
| CIW80_21295 | c0747 | 99.8 | Hypothetical protein YleA |
| CIW80_21975 | c0879 | 99.6 | Hypothetical transcriptional regulator YbiH |
| CIW80_23295 | c0345 | 97.6 | Hemagglutinin |
| CIW80_23810 | c1305 | 99.4 | Minor curlin subunit precursor |
| CIW80_24245 | c1477 | 99.7 | spermidine/putrescine ABC transporter ATP-binding protein PotA |
| CIW80_25525 | c1784 | 99.6 | Hypothetical ABC transporter permease protein YcjP |

**SUPPLEMENTARY TABLE S3C – protein coding sequences conserved in *E. coli* strains CFT073, NCTC12241 and ABU_83972 and truncated in strain Nissle 1917.**

| **Nissle locus tag** | **Matching CFT073 locus tag** | **Alteration in Nissle** | **Annotation** |
| --- | --- | --- | --- |
| CIW80_00330 | c1900 | Truncated | Respiratory nitrate reductase 2 alpha chain |
| CIW80_00705 | c1969 | Truncated | Hypothetical metabolite transport protein YdfJ |
| CIW80_02555 | c2282 | Truncated | Hypothetical protein YecE |
| CIW80_02995 | c2374 | Truncated | Hypothetical protein YedQ |
| CIW80_09000 | c3664 | Truncated | hypothetical protein |
| CIW80_02555 | c2282 | Truncated | DUF72 domain-containing protein |
| CIW80_12230 | c4325 | Truncated | Hypothetical protein YhiV |
| CIW80_13630 | c4681 | Truncated | Ribose operon repressor |
| CIW80_13640 | c4682 | Truncated | Hypothetical transport protein YieO |
| CIW80_19175 | c0322 | Truncated | Putative oligogalacturonide transporter |
| CIW80_09850 | c3824 | 7 residue insertion in Nissle sequence | Hypothetical protein YqjI |
| CIW80_16080 | c5238 | 5 residue deletion in Nissle sequence | class C beta-lactamase |
